# Evaluation of assays for drug efficacy in a three-dimensional model of the lung

**DOI:** 10.1007/s00432-016-2198-0

**Published:** 2016-07-16

**Authors:** Julia M. Huber, Arno Amann, Stefan Koeck, Edith Lorenz, Jens M. Kelm, Petra Obexer, Heinz Zwierzina, Gabriele Gamerith

**Affiliations:** 1 University Hospital for Internal Medicine V (Hematology and Oncology), Medical University of Innsbruck, Anichstr. 35, 6020 Innsbruck, Austria; 2 University Hospital for Pediatrics II, Medical University of Innsbruck, Anichstraße 35, 6020 Innsbruck, Austria; 3 InSphero AG, Wagistr. 27, 8952 Schlieren, Switzerland; 4 Tyrolean Cancer Research Institute, Innrain 66, 6020 Innsbruck, Austria; 5 Oncotyrol, Innrain 66, 6020 Innsbruck, Austria

**Keywords:** Three-dimensional model, Lung cancer, Drug testing, Hanging-drop, In vivo mimicking

## Abstract

**Background:**

The focus of the outlined work is the establishment of a three-dimensional lung model for various drug-screening applications.

**Methods:**

The non-small cell lung cancer (NSCLC) cell line Colo699 was cultivated as monolayer (2D) on plates for 5 days or as microtissues (3D) using a hanging-drop system for 5 and 10 days. Cells and microtissues were treated with afatinib (10–80 µM), cisplatin (100–800 µM) or vinorelbine (25–200 µM) for 24 or 48 hours (h). Cell proliferation and viability were analysed by intra-cellular adenosine triphosphate (ATP) and lactate dehydrogenase release (LDH) assays, annexin V/propidium iodide (PI) staining, and cell cycle determination. Microtissue morphology and size, as well as cell death were evaluated via phase contrast microscopy.

**Results:**

Our results demonstrate the valid determination of viability and cell death using established assays in the 3D system for drug testing. The comparison of ATP, LDH and cytometry data showed moderate (0.40) to very strong (0.99) correlations. Thereby, we observed partially significant differences in drug efficacy between microtissues and 2D cultures dependent from the applied treatment and read-out method. Altogether, microtissues developed resistance to cisplatin and vinorelbine; but remained more vulnerable to afatinib. These findings were confirmed with microscopy.

**Conclusion:**

In summary, we established an NSCLC 3D test system with multiple assays compatible for drug-testing applications of substances with different mechanisms of action. In addition, our data support the usage of microtissues as more accurate tools for drug-efficacy testing with the possibility of long-term cultivation and treatment.

**Electronic supplementary material:**

The online version of this article (doi:10.1007/s00432-016-2198-0) contains supplementary material, which is available to authorized users.

## Introduction

The progress from drug discovery to its commercialization is complex, lengthy and expensive (Suggitt and Bibby 2005). Currently, a drug candidate that enters Phase I trials will successfully proceed further with a probability of just 8 %. This fact urges the need for quantitative and physiologically relevant cell culture in vitro systems better resembling in vivo conditions. Therefore, new in vitro methods should be integrated in the drug-screening process (Pampaloni et al. 2007; Singh et al. 2015). A growing body of research is contributing to the use of three-dimensional (3D) tissue models to close the gap between two-dimensional (2D) cell culture and animal models (Edmondson et al. 2014).

In vitro systems have the advantage to minimize ethical issues and to be more cost-effective compared to in vivo models. Traditionally, in vitro drug testing and screening are conducted on cultured monolayer cells and, therefore, cannot reflect complex response to cytotoxic agents, targeted therapeutics (e.g., tyrosine kinase inhibitors, TKIs) and antibodies at tissue levels (Phung et al. 2011). This paper describes a novel approach to test in vitro toxicity in a microtissue setting, where microtissues of a defined size and shape are formed by seeding equal counts of cells in hanging-drop plates. Cells cultivated on plastic surfaces expected to exhibit an increased sensitivity to cytotoxic drugs, while compounds targeting cell–cell adhesions, cell maturation, epithelial mesenchymal transition (EMT) and stemness features often show a decreased efficacy in 3D cell culture (Amann et al. 2014). The in vivo-like microenvironment of in vitro microtissues is the result of numerous factors including the formation of molecular gradients within the microtissues (e.g., nutrients, oxygen, growth factors, as well as metabolites and paracrine factors) (Thoma et al. 2014; Achilli et al. 2012). In addition, microtissues develop an in vivo-like microenvironment by forming more complex cell-to-cell interactions and cell-to-ECM adhesions. These serve as additional signals, including mechanical forces and biochemical signals, that can influence cell shape, motility, proliferation, differentiation, as well as gene expression (Pampaloni et al. 2007; Achilli et al. 2012; Asthana and Kisaalita 2012; Lovitt et al. 2014). Thus, 3D cell culture models reflect in vivo tumour growth more reliably and can provide better read outs for drug testing (Amann et al. 2014).

To evaluate the effect of cytotoxic/cytostatic substances on this three-dimensional arrangement of cells, we investigated cells for their viability and proliferation. There are different methods available to verify cellular conditions of cells cultivated in a 2D setting. To investigate which of these assays are compatible to quantify proportions of viable, apoptotic, necrotic and proliferating cells in microtissues, we adapted a panel of commercially functional assays for the advanced screening of cells in 3D microtissues. The cell viability in a particular experiment can be illustrated by measuring: (1) adenosine triphosphate (ATP) levels (Crouch et al. 1993), (2) the integrity of cellular membranes and (3) the activity of cellular enzymes, such as lactate dehydrogenase (LDH) (Chan et al. 2013). To test for the validity of assay results,(4) flow cytometry analyses using annexin V and propidium iodide were performed. To differentiate between cytotoxic and cytostatic effects, we additionally performed (5) cell cycle analyses. The confirmation of all results was performed by light microscopy of microtissues. The focus of the outlined work is to test the compatibility of different assay systems to investigate viability and proliferation as well as the analysis and comparison of the differential response of the NSCLC cell line Colo699 cultivated in 2D and 3D to various anticancer therapeutics: Afatinib (BIBW 2992), as a representative of the current generation of TKIs, which irreversibly targets EGFR/HER2 (ErbB1/EGFR^wt^, EGFR^L858R^, EGFR^L858R/T790M^, ErbB2/HER2, and ErbB4), cisplatin, an inhibitor of DNA replication, and vinorelbine which prevents microtubuli polymerization, and, therefore, inhibits mitosis. We established a quantitative and qualitative method for the characterization of tumor cell viability and morphology of cells in 2D and microtissues, respectively.

## Materials and methods

### Reconstitution and storage

Afatinib, cisplatin and vinorelbine (Selleckchem) were reconstituted in DMSO and stored as aliquots at −80 °C until use. Predilutions in DMSO and dilutions in medium were performed immediately before use. RNase A (Sigma) was reconstituted in A. dest. at a concentration of 10 mg/ml, Propidium Iodide (Sigma) was dissolved in A. dest. at a concentration of 1 mg/ml.

### Cell culture

The human non-small cell lung cancer cell line Colo699 (DSMZ, ACC196) was used. For 2D culture, cells were cultivated as monolayer in DMEM low glucose (Lonza) supplemented with 10 % fetal calf serum (FCS) (Sigma-Aldrich, Munich, Germany, Lot 010M3396) and 100 U/ml penicillin, 100 mg/ml streptomycin solution, and 2 mM l-glutamine (PAA). Cells were cultivated at 37 °C in a humidified 5 % CO_2_-containing atmosphere. For cytotoxicity studies, cells were either cultivated in 12-well plates at a density of 45,000 cells per well or in 96-well plates at a density of 5000 cells per well, respectively for 5 days.

### Microtissue culture

For the production of 3D cultures, the Gravity-PLUS™ microtissue culture system (InSphero AG, Zurich, Switzerland) was used. The cultivation strategy was performed as described earlier (Amann et al. 2014) comprising the following modifications: after cells had been grown subconfluently in cell culture plates (Falcon), they were seeded into the hanging-drop plates. Therefore, cells were washed once with PBS and detached with Accutase (PAA) for 5 min at 37 °C. Thereafter, the enzymatic reaction was stopped with addition of cell culture medium. Then, cells were counted and seeded in 40 µl drops at a density of 2500 cells/drop. Cells were allowed to aggregate for 5 days.

### Cell and microtissue treatments

After 5 days of cultivation, half of the initial volume (20 µl from the microtissues; 50 µl of the 2D wells) was aspirated and fresh medium containing cisplatin (final concentration: 800, 400, 200 and 100 µM), vinorelbine (final concentration: 200, 100, 50 and 25 µM), or afatinib (final concentration: 80, 40, 20, and 10 µM) was added, respectively. Control microtissues and cells were treated with the corresponding amount of DMSO which was denoted as vehicle in the outlined work. Assays were performed after 24 and 48 h of treatment. Therefore, the cells and supernatants were inserted in the assays stated below.

### Supernatant harvesting

The supernatants of four 3D microtissues (4 × 20 µl) and two 2D wells (2 × 75 µl) were pooled for each substance concentration to be inserted into the outlined assays (Table 1) mentioned below. The remaining medium per well of the 96-well plate was 25 µl and the remaining medium per capillary of the microtissue plate was 20 µl, respectively.

**Table 1:** Correlation of ATP and LDH assays with flow cytometry

Cultivation (duration)	24 h Treament					
	Afatinib		Cisplatin		Vinorelbine	
	ATP	LDH	ATP	LDH	ATP	LDH
2D (5 days)	0.865	−0.782	0.966**	−0.333	0.957*	−0.933*
3D (5 days)	0.996**	−0.993**	0.988**	0.729	0.939*	−0.987**
3D (10 days)	0.996**	−0.991**	0.971**	−0.425	0.717	−0.770

Cultivation (duration)	48 h Treatment					
	Afatinib		Cisplatin		Vinorelbine	
	ATP	LDH	ATP	LDH	ATP	LDH
2D (5 days)	0.877	−0.550	0.952*	−0.655	0.879*	−0.845
3D (5 days)	0.937*	−0.929*	0.942*	−0.865	0.961**	−0.965**
3D (10 days)	0.985**	−0.995**	0.943*	−0.769	0.909*	−0.919*

Assay results of intra-cellular ATP and LDH release are correlated with data gained from cytometry analysis-based viability data

* Correlation is significant at the 0.05 level (2-tailed)

** Correlation is significant at the 0.01 level (2-tailed)

**Table:** 

Assay type	Volume/assay [µl]
CellTiter-Glo luminescent cell viability assay	25
CytoTox-ONE™ Homogeneous Membrane Integrity Assay	20

### Determination of ATP content (intra-cellular)

The commercially available CellTiter-Glo^®^ luminescent cell viability assay kit (Promega) was used with the following modifications: To quantify intra-cellular ATP, 25 µl CellTiter-Glo was added to the remaining cells in the 96-well plate and 20 µl CellTiter-Glo was added to the remaining microtissues in the capillaries. The cells and microtissues were then incubated at room temperature for 30 min. The detection of the luminescence signal was performed in a white microtiter plate using a VICTOR X5 2030 Multilabel Reader (Perkin Elmer, MA, USA).

### Determination of LDH release (extra-cellular)

The commercially available CytoTox-ONE™ Homogeneous Membrane Integrity Assay (Promega) was performed with the following adaptations: to quantify LDH release 20 µl substrate was added to 20 µl supernatant and incubated for 10 min at room temperature. The enzymatic reaction was stopped using 10 µl stop solution.

### Determination of LDH retention (intra-cellular)

Cell and microtissue cultivation, treatment and collection of supernatant of the cells and microtissues were performed as described in the above section. For quantification of the intra-cellular LDH content, the commercially available CytoTox-ONE™ Homogeneous Membrane Integrity Assay (Promega) was performed with the following modifications: 20 and 25 µl lysis solution (cell culture medium/2 % Triton-X100) was added to 20 µl of the remaining supernatant of the microtissues and 25 µl of the remaining supernatant of the cells, respectively. After addition of lysis solution, a final concentration of 1 % Triton-X100 was achieved. Complete lysis of cells and microtissues was achieved after an incubation of 5 h at 37 °C. The quantification of the LDH content in the supernatant was depicted in the section above.

### Apoptosis detection by annexin V/propidium iodide

At least four microtissues were pooled in one tube and washed with PBS twice. After complete buffer removal, microtissues were immersed in 300 µl Accumax (Millipore) and incubated for 30 min at 37 °C while vortexing after 15 min. The cells cultivated in 12-well plates were detached using accutase (PAA, Pasching, Austria). The enzymatic reaction was stopped with 1 ml complete medium. The cells were centrifuged and washed with PBS. The cell pellet was then resuspended in 200 µl annexin binding buffer, including 2 µl annexin APC (BD, #550475) and 1 µl propidium iodide (50 µg/ml). For the analyses on a FACS Calibur (BD), at least 10,000 cells were captured and evaluated using FlowJo v10.

### Cell cycle determination

Cell and microtissue harvesting and cell preparation was performed as in the apoptosis detection protocol. Cell cycle determination was adjusted from the original protocol from Riccardi and Nicoletti (2006). Therefore, cells were centrifuged and resuspended in 1 ml PBS. Fixation of cells was performed by addition of 2.5 ml ethanol while vortexing the cell suspension. Cells were incubated at −20 °C overnight. Afterward, they were incubated with 250 µl staining buffer (0.05 % Triton X-100, 0.05 mg/ml propidium iodide, 0.1 mg/ml RNaseA) for 40 min at 37 °C. After centrifugation and resuspension in with 300–500 µl PBS for cytometry, analyses were performed on a FACS Calibur (BD) where at least 8000 cells were captured and evaluated using FlowJo v7.6.5.

### Light microscopic assessment

Morphological appearances of the Colo699 microtissues and their structural changes after four, 24 and 48 h of treatment by afatinib, cisplatin or vinorelbine were observed using an Olympus IX70 (Japan) inverted light microscope. The images were captured using the ProgRes CapturePro 2.6 software (Jenoptic Germany) at a 400× magnification.

### Statistical analysis

Data were analysed from three independent experiments performed in triplicates by normalizing to fluorescence or luminescence of wells and microtissues containing vehicle-treated cells. Statistical analyses were performed using SPSS Version 22. Global differences in assays were evaluated by an independent sample Kruskal–Wallis test followed by pairwise comparison using the Mann–Whitney *U* test. A *p* value of ≤0.05 was taken as statistically significant. Graphs were prepared using GraphPad Prism Version 5.03. Results were expressed as mean ± standard error (SEM). The statistical relationship between ATP, LDH assays and cytometry analyses was determined by a Kolmogorov–Smirnoff test to prove normal distribution followed by a Pearson correlation analysis.

## Results

We generated Colo699 microtissues using the hanging-drop technology; cells and microtissues were cultivated as follows: 2D for 5 days and 3D for 5 and 10 days. The outlined investigations confirm that assays based on tumor cell microtissues are suitable to indicate and quantify effects of compounds on tumour cell survival and proliferation. To characterize cellular cytotoxicity in greater detail, various assays were performed followed by a flow cytometry analysis to confirm assay results.

### Assessment of viability by intra-cellular ATP (lysate)

**Fig. 1:**
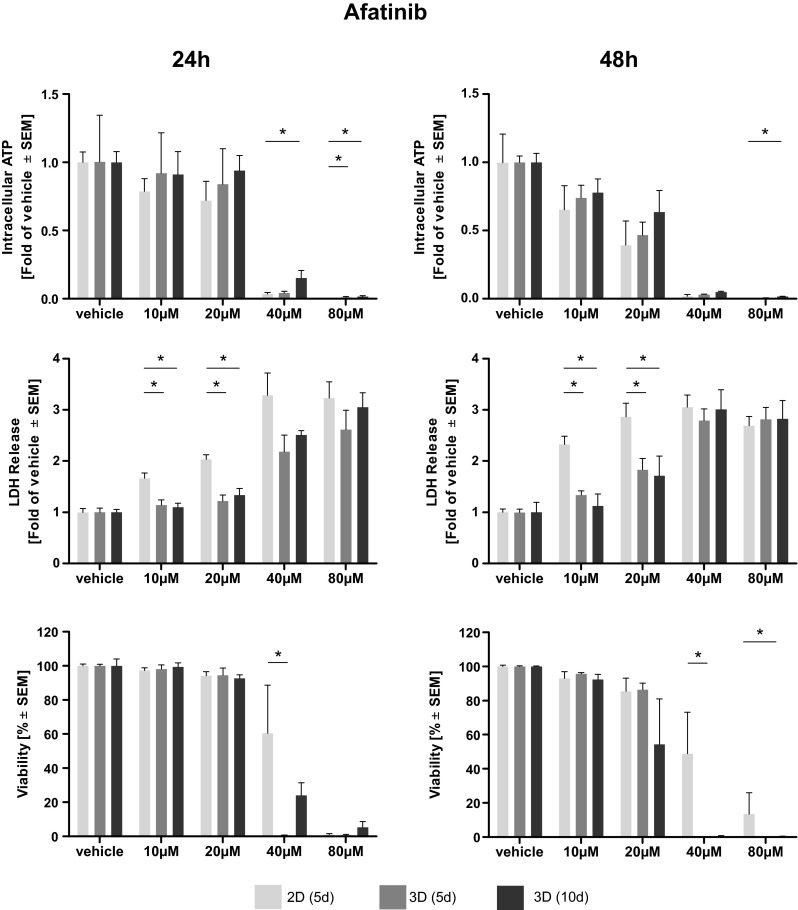
Dose–response curves of afatinib. Cells and microtissues are treated with increasing doses of the tyrosine kinase inhibitor afatinib for 24 and 48 h. Response of 2D cultivated cells and microtissues to afatinib was determined measuring intra-cellular ATP, LDH release, and cell viability (*p < 0.05; *n* = 3 for ATP assay; *n* = 3–7 for LDH assay; *n* = 3–4 for flow cytometry)

**Fig. 2:**
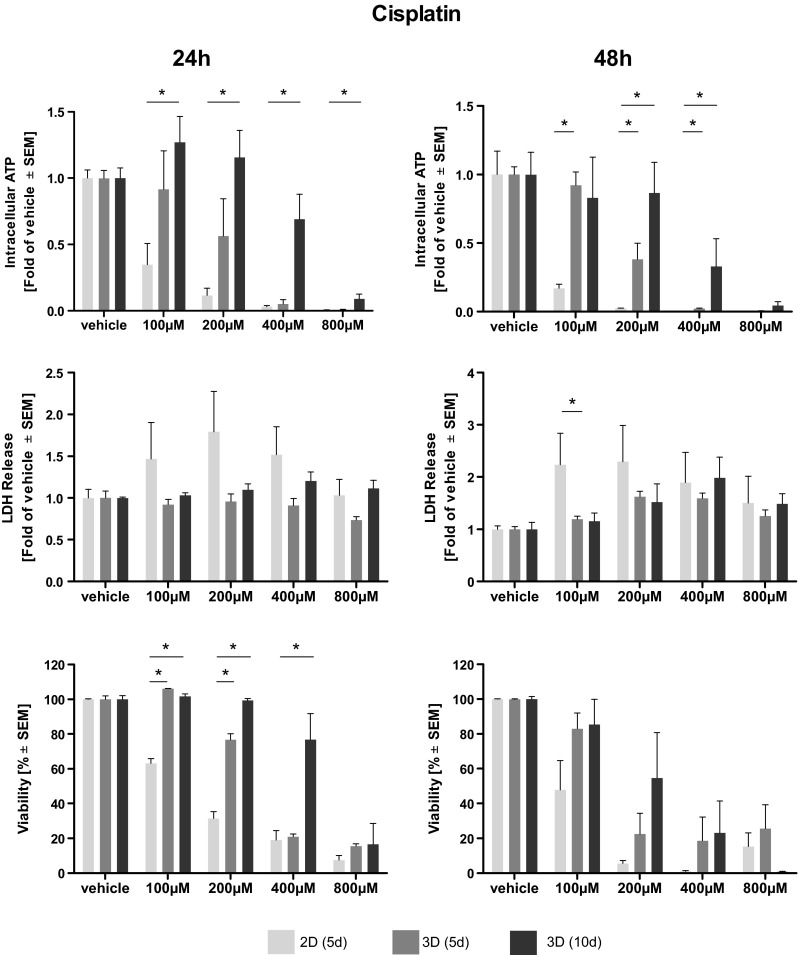
Dose–response curves of cisplatin. Cells and microtissues are treated with increasing doses of the DNA-intercalating molecule cisplatin for 24 and 48 h. Response of 2D cultivated cells and microtissues to cisplatin was determined measuring intra-cellular ATP, LDH release, and cell viability (*p < 0.05; *n* = 3 for ATP assay; *n* = 3–7 for LDH assay; *n* = 3–4 for flow cytometry)

**Fig. 3:**
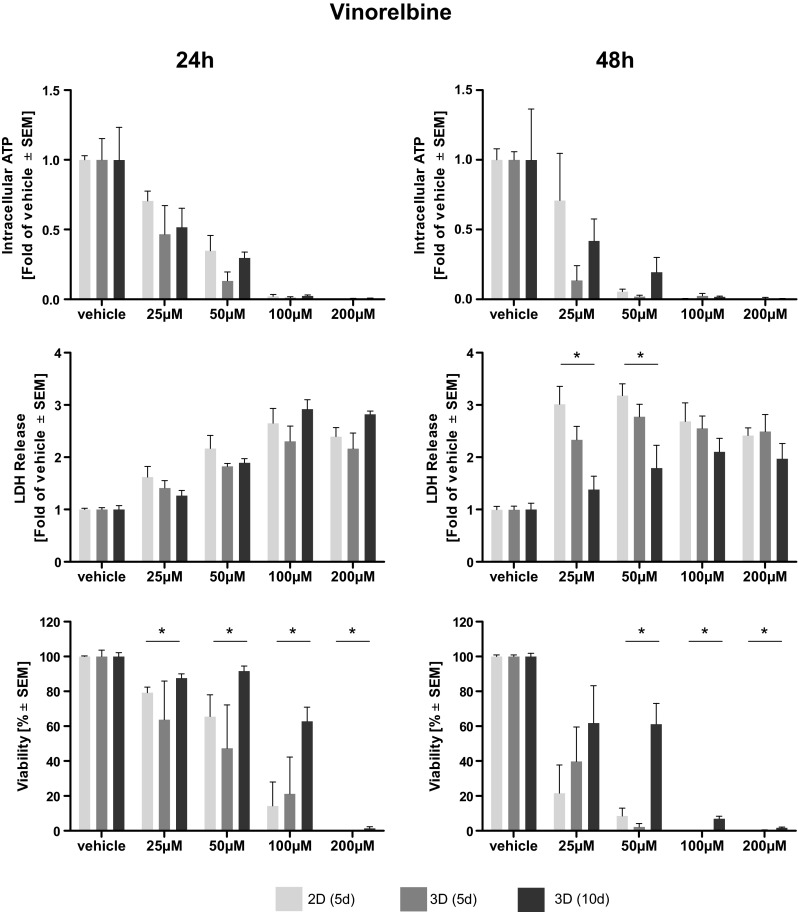
Dose–response curves of vinorelbine. Cells and microtissues are treated with increasing doses of the anti-mitotic microtubuli inhibitor vinorelbine for 24 and 48 h. The response of 2D cultivated cells compared to microtissues was determined measuring intra-cellular ATP, LDH release and cell viability (*p < 0.05; *n* = 3 for ATP assay; *n* = 3–7 for LDH assay; *n* = 3–4 for flow cytometry)

Afatinib treatment led to a dose-dependent reduction of intra-cellular ATP, an indicator of cell viability. Elevated levels of ATP and, thus, higher viabilities were observed in long-term cultivated microtissues. Statistical significance compared to 2D cultivated cells was reached only at higher doses of Afatinib (40–80 µM) (Fig. 1). A dose-dependent reduction of intra-cellular ATP in microtissue and cell lysates induced by cisplatin treatment was observed. After 24 h of treatment significantly higher levels of ATP, indicating a better viability, were found in cisplatin-treated microtissues cultivated for 10 days compared to cells cultivated in 2D. Two-dimensionally cultivated cells have significantly less intra-cellular ATP after 48 h of treatment even compared to microtissues cultivated for 5 days (Fig. 2). Vinorelbine treatment induced a dose-dependent reduction of ATP in microtissue and cell lysates, which is even more pronounced after 48 h. No significant beneficial effect of extended microtissue cultivation could be verified (Fig. 3).

### Assessment of membrane integrity (LDH)

The membrane integrity was determined measuring LDH release. Afatinib treatment of microtissues and cells led to a general dose-dependent increase of LDH in the supernatant. Significantly elevated levels of LDH were observed in 2D cultivated cells compared to microtissues (5 and 10 days) treated with afatinib at doses of 10–20 µM. This effect was irrespective of their cultivation duration (Fig. 1). Dose-dependent differences in LDH release after cisplatin treatment could hardly be observed after 24 but after 48 h. Solely after treatment with 100 µM cisplatin for 48 h statistical significantly higher LDH release in 2D than in microtissues (Fig. 2). Cells and microtissues responded to increasing vinorelbine doses with increased LDH release. There was no difference in LDH release between cells cultivated in 2D compared to 3D after 24 h of vinorelbine treatment, significantly elevated LDH release from 2D cultivated cells treated with 25–50 µM vinorelbine was observed after 48 h (Fig. 3).

### Flow cytometry analysis

Complete disaggregation of the cells without affecting their viability was crucial for the precise detection of apoptosis of Colo699 microtissues using flow cytometry. In this assay, the microtissues were dissociated using an enzymatic reaction, and the viability of individual cells was preserved after dissociation. Staining with Annexin V-PE and PI solution allowed us to differentiate between apoptotic cells (annexin^+^/PI^−^); late apoptotic cells (annexin^+^/PI^+^); necrotic cells (annexin^−^/PI^+^) and viable cells (annexin^−^/PI^−^). Data of viable cells were included in the following analyses. Afatinib treatment led to a dose-dependent reduction of viable cells cultivated in 2D as well as within microtissues. Significantly less viable cells were observed in microtissues treated with 40 µM compared to cells cultivated in 2D. In general, viability data revealed by flow cytometry lead to the assumption that cells cultivated in 3D are more susceptible to the cytotoxic effect of afatinib (Fig. 1). Exposure of the microtissues and cells to increasing concentrations of cisplatin resulted in reduced viability of the cultures. In general, increased cell viability of cells within microtissues compared to cells cultivated in 2D was observed almost throughout the complete cisplatin dose range, while significance was only reached after 24 h treatment with 100–400 µM (Fig. 2). It seems that microtissues were more resistant to vinorelbine treatment after long-term cultivation. Significantly elevated levels of viable cells were detected in microtissues which were treated with 25–200 µM vinorelbine for 24 h and 50–100 µM for 48 h (Fig. 3).

### Validation and test assay comparison

The differential levels of intra-cellular ATP were quantified in lysates of two-dimensional cultivated cells and microtissues. The ATP levels represent the metabolic activity of the cells and, therefore, could be used as an indicator of viability. As a consequence of cell membrane damage, LDH is released to the cell culture supernatant. The cytoplasmic enzyme LDH has been used as a marker of cell damage, because of its stability in cell culture medium and its easy measurement after leakage out of cells with a compromised membrane. LDH activity is quantified through lactate to pyruvate conversion coupled to a resazurin to resorufin reduction. The amount of the fluorescent product is proportional to the amount of LDH present in the cell culture supernatant (Legrand et al. 1992). Flow cytometry represents a valid instrument to confirm analyses data gained from ATP and LDH assays. The comparison of these three data sets showed moderate-to-strong correlation (Table 1), except for data revealed from LDH release from cells and microtissues treated with cisplatin.

### LDH retention test

**Fig. 4:**
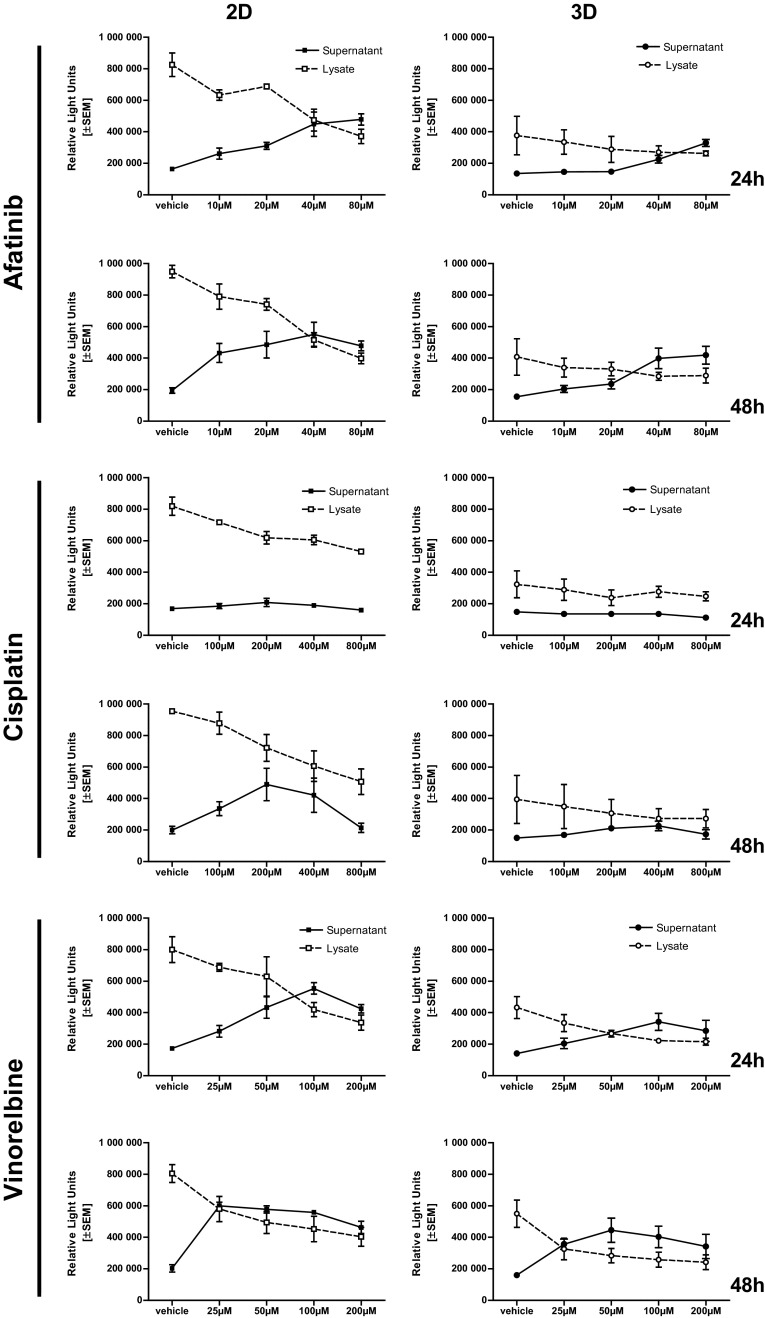
Determination of treatment-dependent LDH retention: afatinib and vinorelbine treatment led to a dose-dependent increase of LDH in the supernatant (*solid line*) simultaneously intra-cellular LDH, released while cells are lysed, decreased (*dashed line*). The LDH retaining effect is observed in cells and microtissues treated with cisplatin

To verify if LDH release is influenced by treatments and to disprove substance-dependent accumulation of LDH within microtissues, we performed lysis of the microtissues and cells with Triton X-100 at a final concentration of 1 % and quantified total LDH. Microtissues and cells treated with vinorelbine and afatinib released LDH nearly completely. Afatinib and vinorelbine treatment led to a dose-dependent increase of LDH in the supernatant simultaneously intra-cellular LDH, released while cells are lysed, decreased. Especially, microtissues treated with cisplatin retain most of the cellular LDH within the cells. Here, the majority of the LDH content is within the lysate of cisplatin-treated cells and microtissues (Fig. 4).

### Cell cycle mediated drug response

**Fig. 5:**
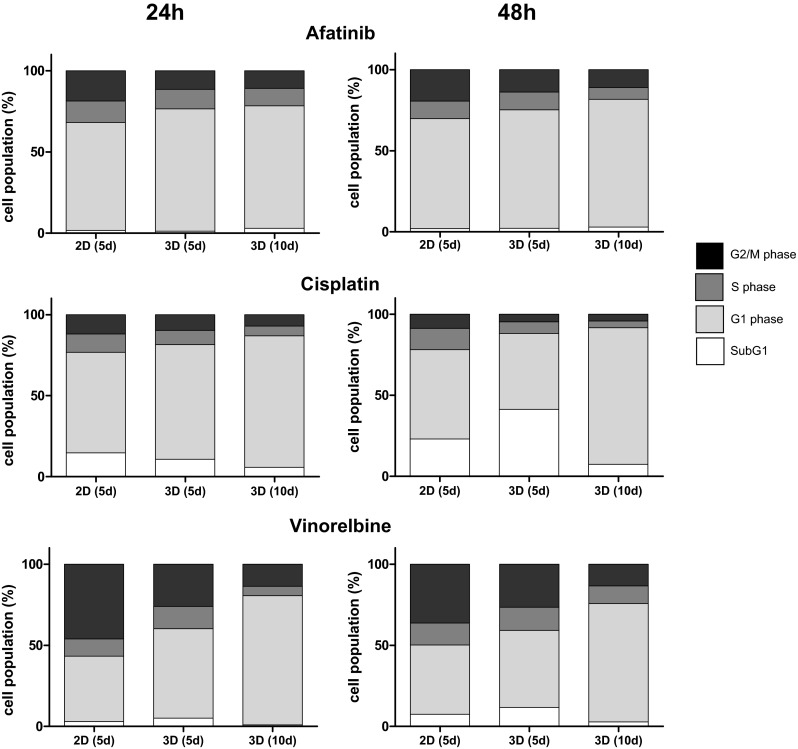
Determination of cytostatic effect: cells and microtissues are treated with increasing doses of the tyrosine kinase inhibitor afatinib, the DNA-intercalating molecule cisplatin and the anti-mitotic microtubuli inhibitor vinorelbine for 24 and 48 h. The cytostatic response of 2D cultivated cells compared to microtissues was determined using cell cycle analyses

Since many of the targeted pathways are involved in mitosis (Shapiro and Harper 1999), cell cycle analyses were performed to visualize cytostatic effects of treated cells cultivated in 2D compared to 3D. To examine the potential antiproliferative activity of different established therapeutics and their differential effects on cells cultivated in 2D and 3D, cell cycle progression was examined by flow cytometry of PI-stained cells. Cell cycle analyses were performed using cells treated with sub-lethal doses of the above-mentioned therapeutics (afatinib 10 µM; cisplatin 200 µM; and vinorelbine 25 µM) (Supplementary Table 1). In microtissues, afatinib treatment led to increased proportions of cells in the G1 phase compared to cells cultivated on plates, while in S- and G2/M phase decreased cell counts are detected. However, the observed differences were solely significant in the G2/M phase between 2D and 3D (5 days) cultivated cells. Treatment with 200 µM cisplatin resulted in a significant decrease of cells in the S- and G2/M phase together with an increase of cells in the G1 phase of cells in microtissues compared to 2D cultivation within the time frame of 24 h. However, reduction of the proportion of microtissue cells within the G2/M phase did not reach significance after 48 h treatment. Treatment with 25 µM vinorelbine led to a significant accumulation of cells in the G1 phase in microtissues cultivated for 10 days compared to 2D cultivated cells. This effect goes hand in hand with a decrease of cells in the S- and G2/M phase (Fig. 5; Supplementary Table 1).

### Light microscopy

Microtissue growth and integrity can easily be visualized by phase-contrast imaging. We observed that microtissues cultivated for 10 days are bigger than those cultivated for 5 days indicating that cells are still proliferating. Afatinib-treated microtissues seem to gain susceptibility to treatment after long-term cultivation, whereas cisplatin and vinorelbine treatment showed a more distinct cytotoxic effect in microtissues cultivated for 5 days compared to those cultivated for 10 days. Treatment with cisplatin or vinorelbine showed a dose- and time-dependent break up of microtissue structures. Surprisingly, microtissues treated with 800 µM cisplatin showed plain edges despite most of the cells are dead as other methods revealed (Supplementary Figure 1).

## Discussion

As NSCLC is associated with high mortality rates and poor clinical performance, an improvement in drug-efficacy testing is still highly warranted. The 2D cell culture models employed in preclinical studies frequently suggest that anticancer drugs have a higher efficacy than is subsequently observed in vivo (Singh et al. 2015). To close this gap, we established a more relevant in vitro system based on a 3D cell culture model of the lung. Based on this model, we assessed the effects of the cytotoxic drugs cisplatin and vinorelbine, as well as the tyrosine kinase inhibitor afatinib on Colo699 cells cultured either in 2D or in a 3D hanging-drop system. The main focus of this project was to validate the use of various established cytotoxicity and viability assays for their use within the hanging-drop microtissues, and to highlight the differences in drug efficacy between the culture systems and the relevance of prolonged culture time.

Similar investigations in microtissues representing other tumor entities, such as osteosarcoma, colorectal, breast or head, and neck squamous carcinoma cell lines underline the importance of improved drug-efficacy testing and the feasibility of 3D drug testing (Singh et al. 2015; Drewitz et al. 2011; Rimann et al. 2014; Onion et al. 2016; Lovitt et al. 2015). In addition, other lung cancer models, such as matrix gels, have been stressed for drug-testing applications by others (Onion et al. 2016; Lama et al. 2013). Based on the results of these studies and our prior work (Amann et al. 2014), we addressed the reproducibility and comparability of results and, especially, assays generated in our 3D system, as most works focused on one read-out method. Therefore, we repeated our tests for several standard drugs, using various complementary cytotoxicity assays (LDH, ATP, Annexin V/PI, and microscopic volume/diameter assessment, as well as cell cycle analyses) within each run. ATP and LDH quantification as well as Annexin V/PI stainings were chosen to cover viability and cell-death assessment. Measurement of intra-cellular ATP, as marker of metabolic activity and cell viability (Chiarugi 2005) and extra-cellular LDH, a marker for membrane integrity and thus measurement for damaged cells, are expected to show the exact opposite demeanor. For vinorelbine, a dose- and time-dependent response was found and a perfect correlation with flow cytometry data achieved. Interestingly, with cisplatin, ATP and flow cytometry showed also a concentration- and time-dependent curve and a high correlation, but with the LDH assay, an intra-cellular accumulation was observed not reflecting the viability of the cells. These findings are in line with Kendig and Tarloff (2007) and support our chosen approach of multi-assay testing to avoid drug/substance induced artefacts. Our data suggest that within the validation process, determination of total intra- and extra-cellular ATP and LDH content is an essential step to demonstrate the influence of therapeutics on ATP and LDH release prior to assay performance. Thus, we summarize that for accurate drug testing, high throughput methods, such as ATP or LDH measurements, after initial validation by light microscopy or flow cytometry provide a robust tool for drug cytotoxicity testing in a 3D model. Our data confirm the importance to include different assays and validation steps in drug testing, as various drugs might affect the read-out of a certain test, thus leading to under- or over-interpretation.

In addition, prior to drug-efficacy testing, the mode of action of a substance needs to be considered, as depicted in the results for vinorelbine, where few cytotoxic effects were measured accompanied by significant increase in cell cycle arrest.

Afatinib, a targeted, cytostatic drug, showed diverse efficacy in 3D versus 2D with a higher vulnerability of the 3D system. Besides, a partially lack of correlation with flow cytometric assays, especially in 2D were observed.

These data underline the need for innovative drug-testing models and the value of flow cytometry and microscopic assessment of microtissue growth in the context of primarily cytostatic, targeted agents. In addition, extended testing with the inclusion of pathway analyses, as shown in the work of Singh et al. (2015), might further improve drug development. Furthermore, patient-derived approaches confirm our results, thus highlighting the usage of these models already in early drug development (Onion et al. 2016).

Besides feasibility and reproducibility, we addressed the question of the ideal time point for drug-efficacy testing in 3D, based on the hypothesis that microtissues mature over time of 3D cultivation accompanied with changes in expression profiles and drug resistance (Lovitt et al. 2014). Therefore, we used the two cultivation times of 5 and 10 days to be compared to 2D-cultures. Our results showed a significant difference between the different periods of cultivation. These findings support the use of extended culture times in drug-efficacy testing in 3D cell culture systems and our system proved superior to others, while long-time cultivation in 2D is not easily achieved.

In summary, we proved the hanging-drop 3D culture of lung cancer cell lines to be an innovative and feasible tool for early drug testing, which might reflect the in vivo situation more appropriately than standard 2D culture systems. The extended cultivation and treatment times will add further impact of the 3D system, in addition to the presence of microenvironmental factors, such as cell–cell interactions and oxygen gradient within the microtissue (Thoma et al. 2014).

## Conclusion

As anticancer drug discovery moves away from the traditional cytotoxic substances to novel, molecular-targeted cytostatic drugs, many emerging new anticancer therapeutics would be considered inactive under the conventional screen. Therefore, it is essential to focus on the integration of innovative drug-screening methods to evaluate the potency of molecular-targeted therapeutics (Choi et al. 2014). Microtissues can reproduce characteristics of avascular tumor nodules and micrometastases of large solid tumors, and better replicate the barrier to drug penetration which is presented by dense tumor tissues (Drewitz et al. 2011; Ma et al. 2012), as well as the nutrient, oxygen and drug gradients (Edmondson et al. 2014). In vitro 3D tissue models are increasingly used to complement 2D cell culture and animal model studies, as an attempt to improve the predictive capabilities of preclinical drug safety and efficacy testing (Amann et al. 2015). The advantage of drug testing using microtissues in hanging-drops is that observation and incubation time can be extended to >5 days (Drewitz et al. 2011; Rimann et al. 2014) compared to a two-dimensional setting and several other 3D culture techniques, where cells reach confluence or lack nutrients and start to die. The implementation of 3D cell culture represents a tool to test cellular responses and influences of anticancer drugs in an in vivo mimicking system (Asthana and Kisaalita 2012; Drewitz et al. 2011; Takagi et al. 2007). Nevertheless, our work underlines the importance to consider the mechanism of action of a new substance, as well as the usage of different assays to validate findings and exclude artificial findings. Furthermore, a sufficient cultivation time is needed to form mature spheroids for drug-testing applications.

In summary, we provide evidence that validated assays which are used to illustrate the constitution of cells in a two-dimensional setting can also be utilized for tumor microtissues, but have to be adjusted to the mechanism of action and validated with different methods before high throughput approaches with single assays are run. Moreover, we demonstrate that cells in a three-dimensional context respond in a different way as compared to cells cultivated on plates, thus bridging the gap between in vitro and in vivo findings.

## Electronic supplementary material

Below is the link to the electronic supplementary material.

Supplementary Fig. 1 (DOCX 363 kb)

Supplementary Table 1 (DOCX 183 kb)
