# Supplementary material for: Evaluation of assays for drug efficacy in a three-dimensional model of the lung
Source: J Cancer Res Clin Oncol. 2016 Jul 16;142(9):1955–66. doi: 10.1007/s00432-016-2198-0 (PMC4978763; doi:10.1007/s00432-016-2198-0)
Supplement: Supplementary file 1 — Supplementary Fig. 1 (DOCX 363 kb) [file 432_2016_2198_MOESM1_ESM.docx]

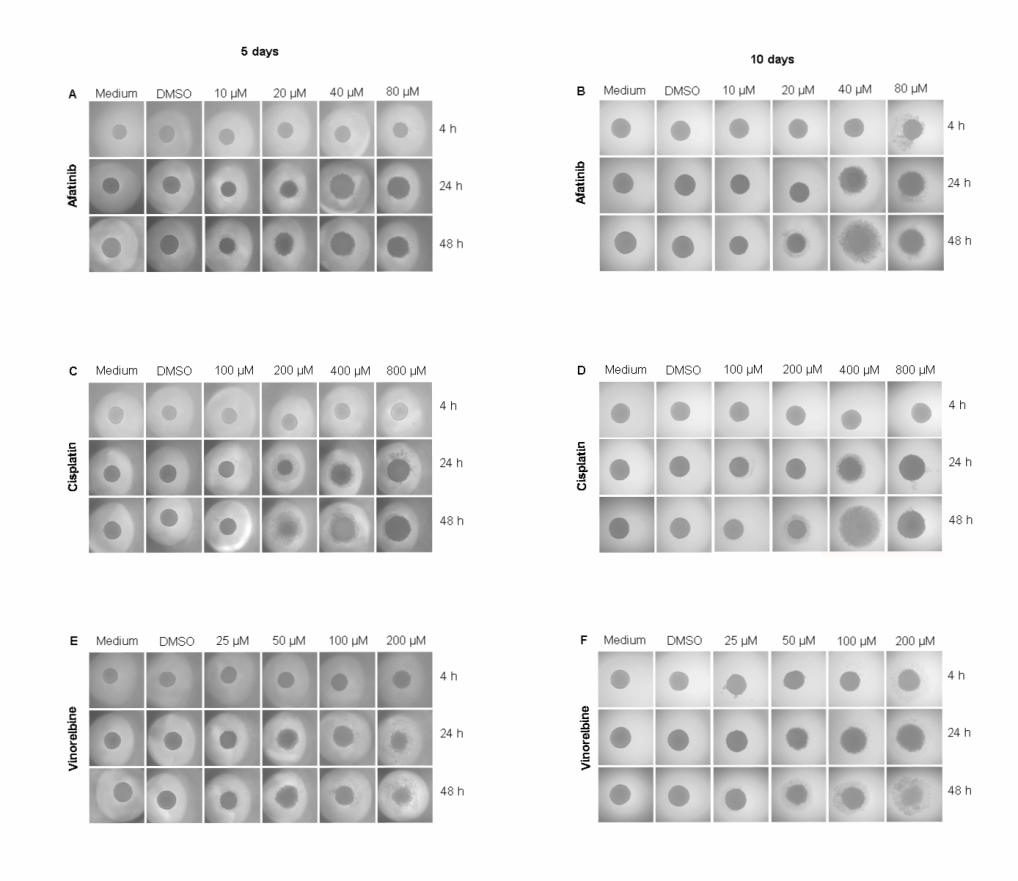


**Supplementary Figure 1: Light Microscopy Assessment of Microtissues:** After cultivation for five (A, C, E) and ten (B, D, F) days, microtissues were treated with afatinib (10 – 80 µM), cisplatin (100 – 800 µM) or vinorelbine (25 – 200 µM), respectively for 4, 24 or 48 hours. Photographs were acquired at a 400 x magnification.
