# Supplementary material for: Evaluation of assays for drug efficacy in a three-dimensional model of the lung
Source: J Cancer Res Clin Oncol. 2016 Jul 16;142(9):1955–66. doi: 10.1007/s00432-016-2198-0 (PMC4978763; doi:10.1007/s00432-016-2198-0)
Supplement: Supplementary file 2 — Supplementary Table 1 (DOCX 183 kb) [file 432_2016_2198_MOESM2_ESM.docx]

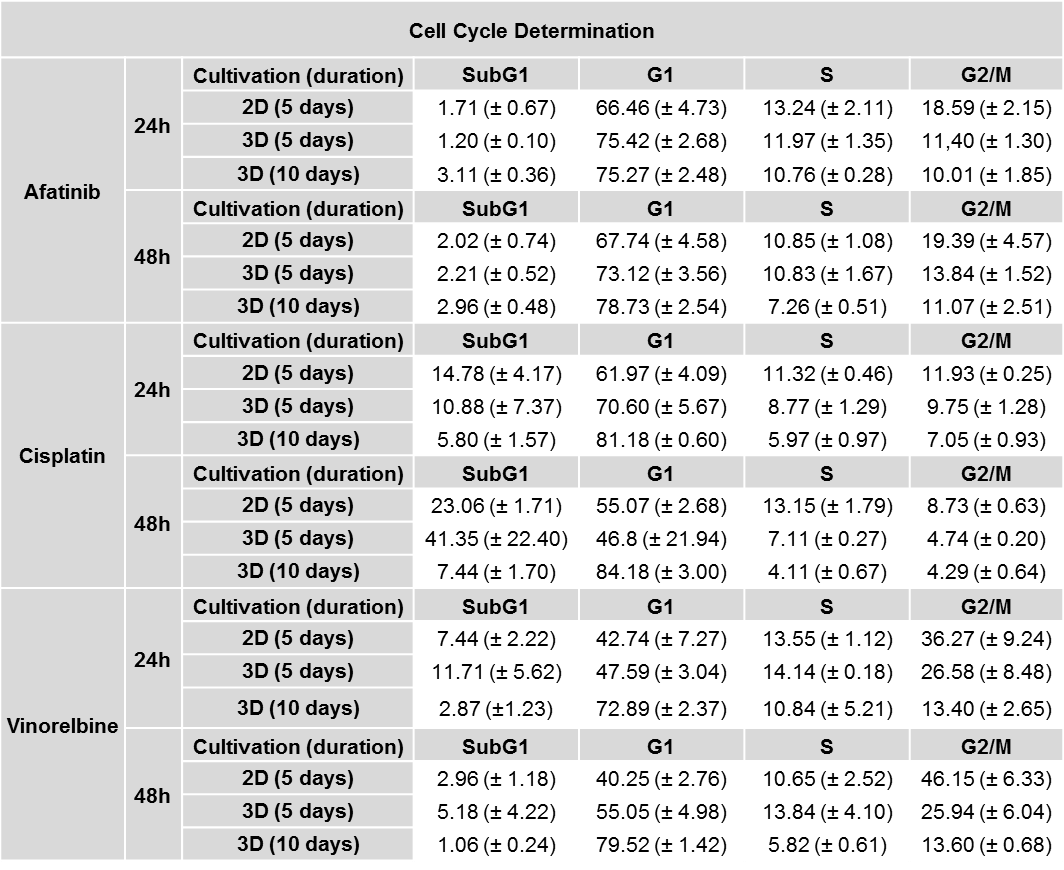


**Supplementary Table 1: Data of Cell Cycle Analyses:** Cells and microtissues are treated with increasing doses of the tyrosine kinase inhibitor afatinib, the DNA-intercalating molecule cisplatin and the anti-mitotic microtubuli inhibitor vinorelbine for 24h and 48h. The cytostatic response of 2D cultivated cells compared to microtissues was determined using cell cycle analyses data are displayed as mean ± s.e.m. (n =3).
